# Nivolumab-Induced Lichenoid Eruption: A Case Report

**DOI:** 10.7759/cureus.86862

**Published:** 2025-06-27

**Authors:** Hugo J Leme, José Ramos, António Magarreiro-Silva, Ana Gouveia, João Alves

**Affiliations:** 1 Dermatology, Hospital Garcia de Orta, Almada, PRT

**Keywords:** cutaneous malignant melanoma, cutaneous oncology, immune checkpoint inhibitors, lichenoid drug eruption, nivolumab-related adverse events

## Abstract

Immune checkpoint inhibitors, such as nivolumab, are associated with a broad spectrum of cutaneous immune-related adverse events, among which lichenoid eruptions are increasingly recognized. We describe a case of nivolumab-induced lichenoid eruption in a patient with metastatic desmoplastic melanoma. Five months after treatment initiation, the patient developed pruritic violaceous papules and plaques, with histopathology revealing lichenoid interface dermatitis. This lichenoid reaction was managed with clobetasol ointment, low-dose oral prednisolone, intralesional corticosteroids, and acitretin, allowing the continuation of immunotherapy with nivolumab, which was completed after one year of treatment.

## Introduction

Immune checkpoint inhibitors, such as nivolumab, a monoclonal antibody targeting PD-1 receptors, have been shown to significantly improve survival outcomes in patients with advanced malignancies, including metastatic melanoma [1]. Despite their therapeutic benefits, immune checkpoint inhibitors are associated with a spectrum of immune-related adverse events, with cutaneous manifestations being the most frequently observed [2]. The incidence of related lichenoid eruption is probably underestimated due to its sporadic publication [2].

We present a case of a nivolumab-induced lichenoid eruption in a patient with desmoplastic melanoma, highlighting diagnostic and therapeutic considerations in the context of a significant management challenge.

## Case presentation

A 74-year-old man with stage IV desmoplastic melanoma of the parietal region (BRAF wild-type) was started on nivolumab. His past medical history included arterial hypertension, dyslipidemia, benign prostatic hyperplasia, and gastroesophageal reflux disease. His chronic medications (fluoxetine, simvastatin, fosinopril, tamsulosin, and omeprazole) had remained unchanged for over five years.

**Figure 1:**
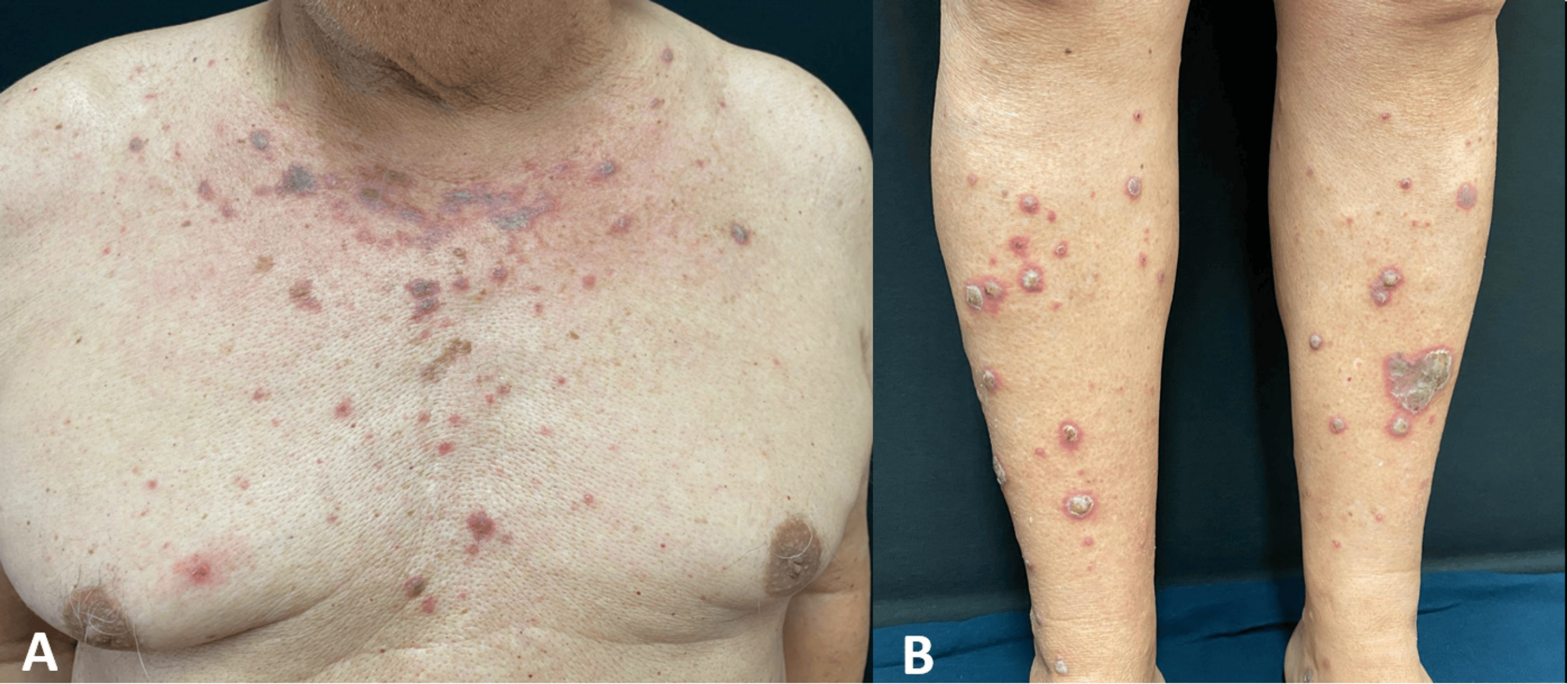
Multiple erythematous to violaceous papules and plaques, some of which were hypertrophic, located on the neckline (A) and lower limbs (B). The distribution and morphology of the lesions are consistent with a lichenoid drug eruption

**Figure 2:**
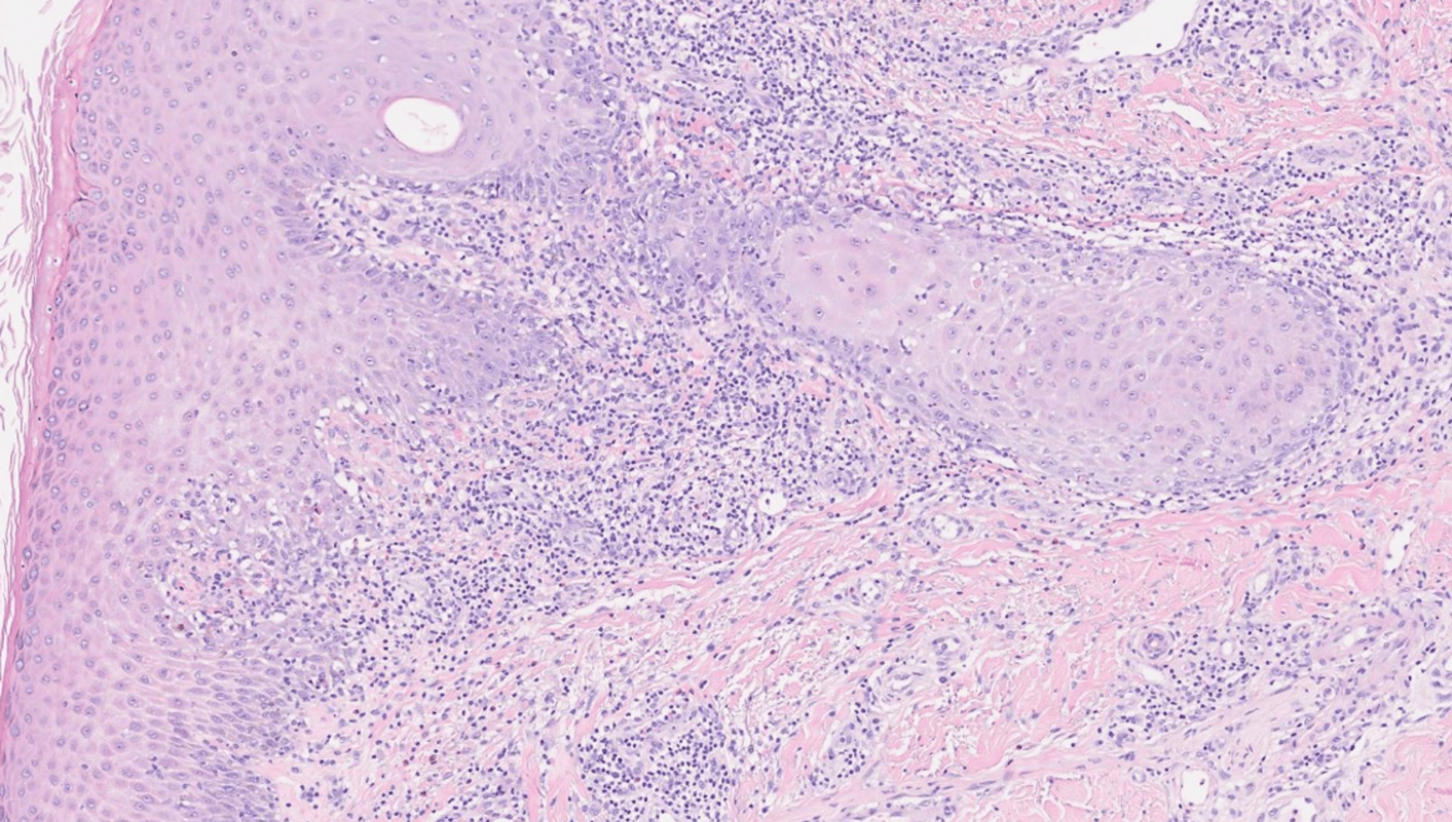
Irregular acanthosis with ortho- and parakeratotic hyperkeratosis. In the superficial and mid-dermis, there is a dense perivascular and interstitial band-like inflammatory infiltrate with interface dermatitis (H&E, ×15)

Approximately five months after initiating nivolumab therapy, the patient was referred to the Dermatology department for the evaluation of a pruritic skin eruption of one month's duration. The lesions were predominantly distributed on the neckline and limbs. Physical examination revealed hypertrophic erythematous to violaceous papules and plaques (Figure 1), without involvement of the mucous membranes, scalp, or nails. Laboratory workup, including complete blood count, renal and liver function tests, and serologies for HIV and hepatitis B and C, was unremarkable. A skin biopsy showed features consistent with lichenoid interface dermatitis (Figure 2). Based on clinical and histopathological findings, a diagnosis of nivolumab-induced lichenoid drug eruption was made. Nivolumab was identified as the most likely culprit drug from a review of our patient's medications at the time of his clinical presentation, based on temporal correlation and exclusion of other potential causes. The patient had no prior history of lichen planus or other lichenoid eruptions.

Initial management included clobetasol ointment twice daily, low-dose oral prednisolone (10 mg/day for one month), and intralesional triamcinolone acetonide (10 mg/mL) for more resistant and hypertrophic lesions on the lower limbs. Higher corticosteroid doses were avoided to preserve the antitumor efficacy of immunotherapy. Approximately eight months after nivolumab initiation, the patient reported symptomatic improvement and continued nivolumab treatment. However, two months later, pruritus worsened and new lesions appeared on the dorsum of the hands, without any changes in nivolumab dosing or signs of melanoma progression. Acitretin was initiated at 10 mg/day and increased to 25 mg/day after one month. At that time, nivolumab was suspended after completing one year of treatment. Three months after discontinuing immunotherapy, the patient showed clinical improvement, with only mild pruritus and persistence of cutaneous lesions under acitretin 25 mg/day. Two months later, the patient presented only with post-inflammatory hyperpigmentation macules, and acitretin was discontinued.

## Discussion

Skin reactions represent the most common adverse effects associated with immune checkpoint inhibitors [3,4], with maculopapular eruptions, pruritus, and vitiligo being the most frequently reported manifestations [3].

Cutaneous lichenoid eruptions are a recognized but underreported adverse effect of PD-1 inhibitors such as nivolumab [2]. These manifestations occur more frequently with anti-PD-1/anti-PD-L1 agents than with CTLA-4 inhibitors [4].

The average elimination half-time of nivolumab is approximately 26 days, but the effects of PD-1 inhibitors are likely to last longer. Cutaneous lichenoid eruptions mostly appear in the first months, but they can also become evident only one year after the immunotherapy and persist after it has been discontinued [5].

Although the exact pathogenic mechanisms underlying nivolumab-induced lichen planus remain to be elucidated, excessive T-cell activation triggered by nivolumab is considered a plausible explanation [1,2].

Tumor responses may be associated with the development of cutaneous immune-related adverse events [5]. The development of these adverse effects has been associated with improved response to immunotherapy and prolonged overall survival, supporting its potential role as a favorable prognostic indicator [6].

Although generally manageable with topical therapies, more extensive or refractory cases may require systemic agents, including retinoids [4]. Dupilumab has already been shown to be effective in managing these patients. Although the exact mechanism remains unclear, it has been proposed that dupilumab's inhibition of IL-4 and IL-13 may indirectly downregulate IL-6-driven Th2 responses, which are elevated in patients with lichen planus and may contribute to disease persistence [7].

Coordination between dermatology and oncology teams is crucial to balance the management of immune-related adverse effects with the continuation of oncologic treatment.

## Conclusions

It is imperative to acknowledge the diverse cutaneous adverse events associated with these agents and to evaluate patients on an individual basis, taking into account the severity of manifestations and response to therapy. Early recognition and tailored intervention can prevent unnecessary discontinuation of life-prolonging immunotherapy. In the present case, resolution of pruritus allowed the patient to maintain a satisfactory quality of life, despite persistent cutaneous lesions, which enabled continuation of nivolumab for a total duration of one year. This case also underscores the value of multidisciplinary collaboration and the need for long-term dermatologic monitoring in patients receiving immune checkpoint inhibitors.
